# Sepsis and Subsequent Psychiatric Morbidity: A Nationwide Population-Based Matched Cohort Study, 2008–2019

**DOI:** 10.1097/CCM.0000000000007105

**Published:** 2026-03-23

**Authors:** Hanna Wetterberg, Anton Nilsson, Adam Linder, Maria Lengquist, Attila Frigyesi, Jonas Sundén-Cullberg, Malin Inghammar

**Affiliations:** 1 Infection Medicine, Department of Clinical Sciences, Lund, Lund University, Lund, Sweden.; 2 Epidemiology, Population Studies and Infrastructures (EPI@LUND), Department of Laboratory Medicine, Lund University, Lund, Sweden.; 3 Register-based Epidemiology, Department Translational Medicine, Lund University, Malmö, Sweden.; 4 Department of Infectious Diseases, Skåne University Hospital, Lund, Sweden.; 5 Anaesthesia and Intensive Care, Department of Clinical Sciences, Lund, Lund University, Lund, Sweden.; 6 Department of Intensive and Perioperative Care, Skåne University Hospital, Lund, Sweden.; 7 Center for Infectious Medicine, Division of Infectious Diseases, Department of Medicine Huddinge, Karolinska Institutet, Stockholm, Sweden.; 8 Department of Infectious Diseases, Karolinska University Hospital Huddinge, Stockholm, Sweden.

**Keywords:** mediation analysis, mental disorders, population control, prescriptions, sepsis

## Abstract

**Objectives:**

To quantify the risk of incident psychiatric morbidity after community-acquired sepsis and assess whether new chronic diseases mediate the association.

**Design:**

Nationwide, population-based matched register cohort; hazards estimated with weighted Cox regression.

**Setting:**

Sweden, linking the National Quality Sepsis Registry, National Patient Register, Prescribed Drug Register, and population registers.

**Patients:**

Ten thousand three hundred eight adults (≥ 18 yr) treated in an ICU for sepsis (2008–2019), matched to 155,705 population controls by sex, age, region, and year. Individuals with a psychiatric diagnosis within 5 years or psychotropic medication within 1 year before index were excluded.

**Interventions:**

None.

**Measurements and Main Results:**

The primary outcome, psychiatric event, was first occurrence after index date of either initiation of a psychotropic medication (anatomic therapeutic chemical classification system code N05A, N05BA, N05C, N06A) in the Prescribed Drug Register (capturing prescriptions from primary and specialist care) or a new *International Classification of Diseases*, 10th Edition mood (F3) or anxiety (F4) diagnosis in specialist care. Weighted Cox models balanced baseline covariates. We used a Landmark approach with risk sets at 0–30, 31–90, 91–365 days; 1–3, 3–5, and greater than or equal to 5 years after the index date. Sepsis was associated with increased hazards of psychiatric events vs. matched controls, with the strongest associations in the first year (0–30 d: adjusted hazard ratio [aHR], 6.2 [5.0–7.7]; 31–90 d: aHR, 7.4 [6.5–8.6]; and 91–365 d: aHR, 2.3 [2.1–2.5]) attenuating over time but remaining elevated through 5 years (1–3 yr: aHR, 1.2 [1.1–1.5]; 3–5 yr: aHR, 1.3 [1.1–1.5]; and ≥ 5 yr: aHR, 1.1 [0.9–1.3]). In mediation analyses considering incident chronic diseases, estimates changed little, suggesting that these conditions did not mediate the association.

**Conclusions:**

Patients with sepsis had a higher subsequent incidence of psychiatric events compared with matched population controls, with a persistently elevated risk for at least 5 years. This increased risk suggests that sepsis may have a long-term impact on psychiatric health, warranting consideration of preventive strategies.

**KEY POINTS**

**Question**: This study sought to quantify the risk of incident psychiatric morbidity in patients with community-acquired sepsis and to assess whether new chronic diseases mediate the association.

**Findings**: Across Landmark periods, sepsis was associated with higher hazards of psychiatric events compared with matched controls from the general population, persisting up to 5 years. Incident chronic diseases did not meaningfully mediate the association.

**Meaning**: Patients with sepsis had a higher hazard of psychiatric morbidity, as captured in healthcare data, underscoring the need for mental health screening and follow-up to ensure appropriate care.

Sepsis is a life-threatening organ dysfunction caused by a dysregulated host response to infection (1). Every year, 49 million individuals worldwide develop sepsis, with 20% in-hospital mortality (2, 3). While advancements in acute treatment have lowered mortality rates in Western countries, they have also resulted in a growing population of sepsis survivors. This patient group faces an increased risk of long-term complications, such as functional disabilities, new morbidities, and elevated mortality (4). Beyond these physical consequences, emerging evidence suggests that sepsis patients might also have an increased risk of psychiatric morbidity (5, 6), but its magnitude and time course remain uncertain.

Factors such as severe psychologic stress, pain, respiratory distress, and delirium have been suggested to contribute to a higher risk of psychiatric morbidity (7–10). However, the specific burden of psychiatric morbidity in sepsis patients is less thoroughly explored. Previous studies have reported varying results with nationwide register-based studies reporting incidences of new diagnoses ranging from 3% to 18% (6, 11, 12), while symptom-based clinical cohorts often found higher prevalences (up to 50% with significant depressive symptoms) (5), one longitudinal study showed no change in the point prevalence of depressive symptoms (13). Comparisons are limited by varying sepsis definitions, short follow-up periods, differences in outcome ascertainment (diagnoses vs. prescriptions vs. symptom scales and primary care often not captured), and the absence of matched controls, making it difficult to attribute subsequent psychiatric morbidity specifically to sepsis.

In the present study, we addressed these gaps by investigating whether patients with community-acquired sepsis have a higher risk of psychotropic medication initiation or psychiatric diagnosis compared with a matched control group from the general population. Leveraging national health registers with a validated sepsis identification approach and over 10 years of follow-up, we examined both short-term and long-term psychiatric morbidity, providing a detailed understanding of the mental health risks associated with sepsis.

## METHODS

### Study Population and Design

We conducted a register-based matched cohort study using data from Swedish national healthcare registers to assess the risk of new prescriptions for psychotropic medication or psychiatric diagnoses following sepsis. We identified all patients 18 years old or older in the Swedish Intensive Care Registry (SIR) who were admitted to an ICU from 2008 to 2019 with a diagnosis of community-acquired sepsis. The full definition and *International Classification of Diseases*, 10th Edition (ICD-10) codes are provided in **Supplementary Methods 1** and **Supplementary Table 1** (https://links.lww.com/CCM/H941).

**Figure 1.**
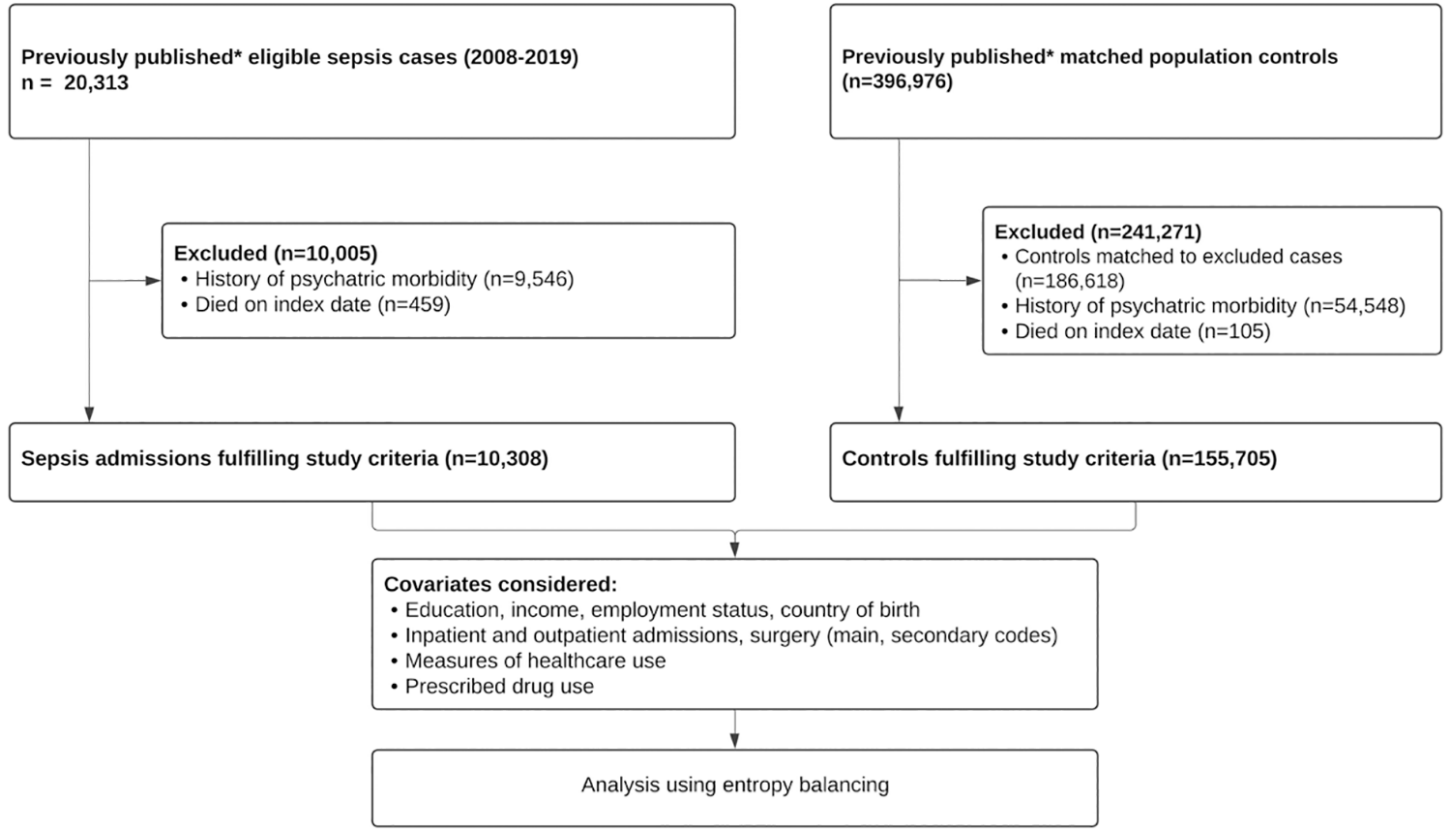
Cohort flow chart for the current study. Starting from the previously published cohorts (20,313 cases with community-acquired sepsis and 396,796 matched controls from the general population matched on age, sex, county of residence, and year of admission [14]), we excluded individuals with a history of psychiatric morbidity, yielding 10,308 sepsis patients and 155,705 controls.

In our previous work, we identified 20,313 adult patients with community-acquired sepsis in the SIR and for each sepsis case, 20 controls from the general population were matched on age, sex, county of residence, and year (14). Controls were sampled without replacement, excluding anyone previously identified as sepsis per the case-definition described in the Supplementary Methods 1 (https://links.lww.com/CCM/H941). For this analysis, we further excluded cases and controls with a history of psychotropic medication prescription based on the anatomic therapeutic chemical classification system (ATC) codes in the Prescribed Drug Register (antipsychotics [N05A], anxiolytics [N05BA], hypnotics and sedatives [N05C], or antidepressants [N06A]) within 1 year before the index date, or psychiatric disease based on the ICD-10 codes in the National Patient Register (NPR; mood disorders [F3], or anxiety disorders [F4]) within 5 years before the index date. We also excluded those who died on the index date (day 0), leaving a final analytic cohort of 10,308 sepsis patients and 155,705 controls. Details of all exclusion criteria and patient flow are provided in **Figure 1**.

We obtained information on covariates and outcomes by linking individual information from other national administrative and health registers with full coverage of Swedish residents: the Longitudinal Integrated Database for Health Insurance and Labour Market Studies-register (information regarding socioeconomic status, mortality and migration, age, sex, area of residence, country of birth, level of education, income sources, and total disposable income per consumption unit), the NPR, covering all inpatient stays and specialized outpatient care, and the Prescribed Drug Register, covering all prescribed medications dispensed at pharmacies.

The study was approved by the Ethical Review Board (Etikprövningsmyndigheten, study title: [Seneffekter och socioekonomiska riskfaktorer vid svår sepsis (Late effects and socioeconomic risk factors in severe sepsis)]; approval date: June 26, 2019; Dnr. 2019-03284), and reporting followed the Strengthening the Reporting of Observational Studies in Epidemiology checklist (**Supplementary Methods 2**, https://links.lww.com/CCM/H941). Since this is a registry-based study, informed consent was waived. All procedures adhered to the standards of the responsible ethics committee and to the 1964 Helsinki Declaration and its later amendments.

### Outcome Definition

The main outcome, psychiatric event, was a composite defined as the first occurrence after the index date of either: 1) initiation of psychotropic medication (ATC codes from the Prescribed Drug Register; antipsychotics [N05A], anxiolytics [N05BA], hypnotics and sedatives [N05C], or antidepressants [N06A]) or 2) a new ICD-10 psychiatric diagnosis of mood (F3) or anxiety disorder (F4) recorded in the NPR (inpatient and specialist outpatient care). As the NPR only covers specialist care and does not include primary care diagnoses, including prescriptions complements case ascertainment as this also captures patients treated in primary care.

### Statistical Analysis

We estimated hazard ratios (HRs) for psychiatric morbidity using Cox regression with robust ses, using time since index (ICU admission date; matched controls were assigned the same date) as timescale. Individuals were censored after the first event (first prescription or diagnosis as defined above), emigration, death, or end of study (December 31, 2019). We applied entropy balancing to control for confounding (15). The covariates considered in the entropy balancing included sociodemographic characteristics (age, sex, county of residence, country of birth, educational attainment, employment, and total disposable income per consumption unit), year, medical history, and measures of healthcare use and prescription drug use (**Supplementary Table 2**, https://links.lww.com/CCM/H941). Missing values were handled by including a “missing” category or set to zero (for income). As the proportional hazards assumption was violated (tested via Schoenfeld residuals), we employed a Landmark analysis approach. Specifically, we defined Landmark time points at 0–30 days, 31–90 days, 91–365 days, greater than or equal to 1–3 years, greater than or equal to 3–5 years, and greater than or equal to 5 years from the index date. At each time point, we rebalanced the baseline covariates, including only individuals who were alive and event-free.

To investigate potential effect modifiers, we stratified predefined subgroups, including sex, age (< 65 vs. ≥ 65), disease severity (measured using the Simplified Acute Physiology Score [SAPS] 3), decision regarding active treatment, and length of ICU stay.

We also performed a mediation analysis to investigate the role of new chronic diseases in the pathway linking sepsis to psychiatric morbidity. The mediators included conditions diagnosed after sepsis but before the psychiatric event, specifically chronic pain syndromes, neurologic complications, cardiovascular complications, respiratory conditions, and renal failure (**Supplementary Table 3**, https://links.lww.com/CCM/H941). To estimate the total effect of sepsis, we first fitted a Cox regression model that included all baseline covariates, as described earlier. Next, we fitted a second weighted Cox regression model, modifying the weighting scheme to account for the specified mediators. Mediators were coded cumulative up to the end of each window and only the first event was included. This second model was used to estimate the direct effect of sepsis on psychiatric morbidity. The difference between the total and direct effects was interpreted as the indirect effect mediated by the included pathways.

All reported *p* values were two-sided and all analyses were performed using Stata, Version 18.0 (StataCorp, College Station, TX) and the *ebalance* package (Jens Hainmueller, Stanford University, Stanford, CA) (15).

## RESULTS

### Descriptives

**Supplementary Table 4** (https://links.lww.com/CCM/H941) displays descriptive characteristics before reweighting of the controls. The median age was 70 (interquartile range [IQR], 60–78) among cases and 69 (IQR 58–76) among controls, with the proportion of females being 38% and 34%, respectively. As expected, sepsis patients had lower socioeconomic status than the unweighted controls and had substantially more comorbidities and higher healthcare utilization.

### Incidence of Psychiatric Events

**Figure 2.**
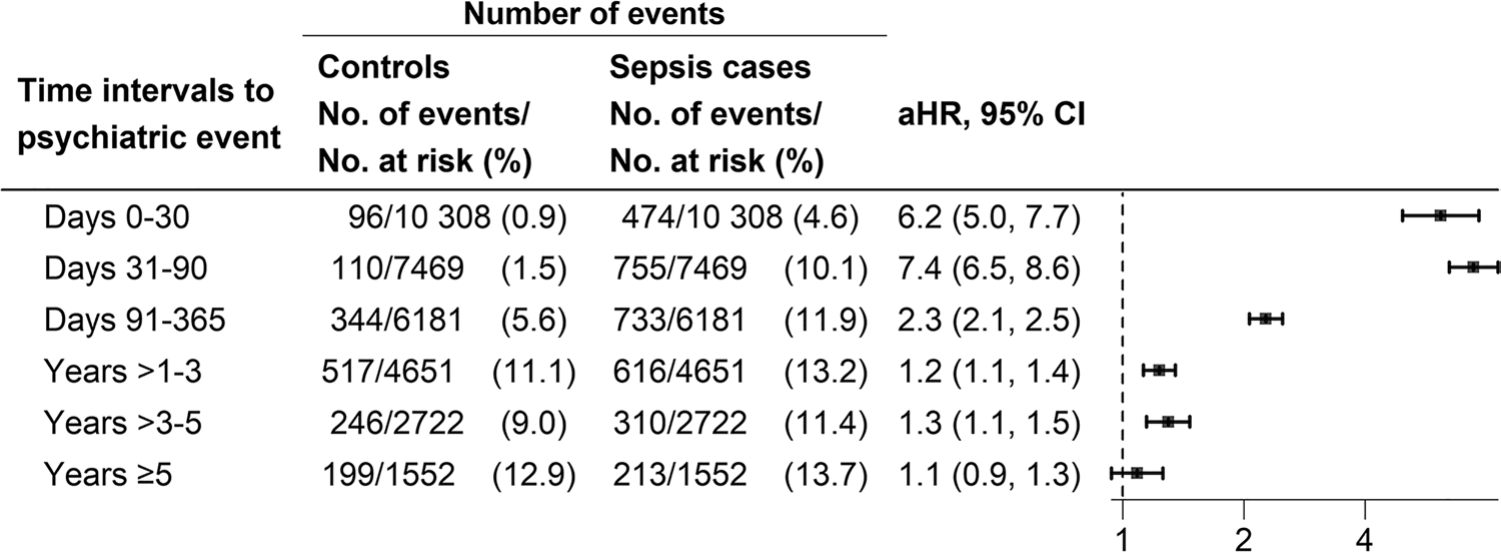
Adjusted hazard ratios (aHRs) for psychiatric events (new prescription or diagnosis) in sepsis cases vs. matched, weighted controls, estimated using Cox regression. Control numbers are rounded. Weights were calculated using individuals eligible for each landmark period. *X*-axis uses a logarithmic scale, *vertical line* at aHR = 1.

Among the 10,308 sepsis patients, the median follow-up was 0.7 years (IQR, 0.1–3.2 yr), resulting in 21,336 person-years of observation. Over this follow-up, 3101 psychiatric events were recorded, corresponding to an incidence rate of 145.3 per 1000 person-years (**Supplementary Table 5**, https://links.lww.com/CCM/H941). In comparison, the weighted controls had a median follow-up of 2.9 years (IQR, 1.2–4.5 yr), yielding 38,472 person-years: 2,634 psychiatric events occurred in this group, with a weighted incidence rate of 68.5 per 1,000 person-years. Kaplan-Meier curves showed that sepsis cases had a higher probability of psychiatric events (log-rank test *p* < 0.001; and **Supplementary Fig. 1**, https://links.lww.com/CCM/H941). Sepsis was associated with an increased long-term risk of psychiatric events compared with the matched control group up to 5 years after the admission date (**Fig. 2**). The incidence was 6–7 times higher in sepsis patients than in controls during the first 90 days after the sepsis event, twice as high during the remainder of the first year, and 20–30% higher up to 5 years after. The absolute risk of event was 5.0 percentage points higher after 31 days, 13.2 percentage points higher after 91 days, and 17–18 percentage points higher during the years 1 to greater than or equal to 5 years after, compared with the weighted controls (**Supplementary Table 6**, https://links.lww.com/CCM/H941).

### Incidence Stratified by Prescription and Diagnosis

Among sepsis cases, 29.7% initiated psychotropic medication, giving an incidence rate of 143.4 per 1000 person-years (**Supplementary Tables** 5 and **7**, https://links.lww.com/CCM/H941). In comparison, 25.2% of weighted controls initiated medication, with an incidence rate of 67.4 per 1000 person-years. The most frequently initiated medication class was hypnotics and sedatives, initiated in 19.9% of cases and 14.4% of weighted controls (Supplementary Table 7, https://links.lww.com/CCM/H941). The difference between cases and controls in the incidence of initiating a psychiatric medication followed the same pattern as the composite outcome, with an increased incidence of 6–7 times during the first 3 months, and then leveling off but remaining increased up to 5 years past index (**Supplementary Fig. 2**, https://links.lww.com/CCM/H941).

When analyzing new psychiatric diagnoses identified by ICD codes in inpatient or specialist outpatient care, 3.3% of cases received a new diagnosis, yielding an incidence rate of 11.3 per 1000 person-years (Supplementary Tables 5 and 7, https://links.lww.com/CCM/H941). In comparison, 2.4% of weighted controls received a new diagnosis, with an incidence rate of 5.4 per 1000 person-years. The most common new diagnosis was anxiety disorders, occurring in 2.0% of cases and 1.5% of controls. The incidence of psychiatric diagnoses was 6–13 times higher in sepsis cases during the first 3 months (Supplementary Fig. 2, https://links.lww.com/CCM/H941). Thereafter, the point estimates varied from 1.4 and 2.3 and were statistically significant in all subsequent time periods.

### Effect Modifiers and Pathways Linking Sepsis to Psychiatric Events

In the moderation analysis, the association between sepsis and psychiatric events was similar between men and women, somewhat stronger in younger age groups between 91 days and 3 years after sepsis, and similar across SAPS 3 levels and treatment decisions, while patients with an ICU stay longer than 7 days had a stronger association during the first year (**Supplementary Fig. 3**, https://links.lww.com/CCM/H941).

Including new chronic diseases yielded lower adjusted hazard ratios (aHRs) but overlapping CIs vs. the main model, suggesting limited mediation and no material explanation of the association between sepsis and psychiatric events (**Supplementary Fig. 4**, https://links.lww.com/CCM/H941).

## DISCUSSION

In this nationwide population-based matched cohort study, we found that patients with sepsis and no recent prior record of psychotropic medication or psychiatric morbidity had an increased risk of subsequent psychiatric events during the first 5 years after sepsis. Using a robust weighting method, the incidence of psychiatric event was more than twice as high during the first year, and 20–30% higher after 1–5 years among patients with sepsis than among matched controls from the general population. At 5 years, the cumulative absolute risk was 43.0% in the sepsis cohort vs. 27.6% in controls. The elevated risk was primarily driven by the initiation of psychotropic medications, particularly hypnotics and sedatives, which were initiated in 19.9% of cases and 14.4% of controls, respectively. We also found that patients with an ICU stay of at least 7 days had a greater increase in risk of psychiatric events during the first year.

Consistent with our findings, several studies have reported an elevated risk of psychiatric morbidity among critically ill patients. A British study found a two-fold increase in psychotropic medication prescribing within the first year for critically ill vs. noncritically ill patients (16). Likewise, a Danish study observed a similar two-fold rise in new psychoactive prescriptions compared with the general population, and a 20% cumulative incidence in the first year (7). A recent Swedish study reported 15.5% of critically ill patients initiating antidepressant within 1 year (9). In our ICU-treated sepsis cohort, the 1-year absolute risk for any psychiatric event was 26%. This higher figure is expected as we use a broader definition of outcome—first occurrence of either psychotropic prescription or a specialist-care diagnosis.

A Canadian study found that 14% of critically ill patients received a new mood or anxiety disorder diagnosis within the first year (17), similar to the findings in a German study in which 12% of sepsis patients received a new depression diagnosis (6). These rates are higher than our finding of 3.3% of sepsis cases receiving a mood or anxiety disorder diagnosis over the entire follow-up period. This discrepancy is likely due to differences in data sources: the Canadian and German studies utilized health claims data, which include hospitalizations as well as outpatient consultations and physician visits, whereas our study relied on inpatient or specialist outpatient care records for diagnostic codes. Consequently, while our study may under-report diagnoses managed in primary care settings, inclusion of the Prescribed Drug Register allows us to capture all prescriptions, providing a comprehensive overview of treated psychiatric illness.

To understand the underlying reasons for the increased risk of psychiatric morbidity observed in sepsis patients, we examined different effect modifiers and new chronic conditions as mediators. The analyses indicated that new chronic diseases did not mediate the relationship between sepsis and subsequent psychiatric morbidity, suggesting that the heightened psychiatric risk is not primarily driven by the emergence of additional chronic health conditions. However, we identified that the relative risk was highest in the group of sepsis patients with a length of ICU stay of at least 7 days, findings similar to a recent study on critically ill patients (9). Beyond being a proxy for severity of illness, an extended ICU stay may amplify psychologic stress and expose patients to traumatic experiences, which can contribute to the development of psychiatric symptoms. Biologically, critical illness can result in systemic inflammation, oxidative stress, and impaired neuroplasticity, all of which have been linked to the development of depression after sepsis (18). Beyond this, autonomic and hypothalamic-pituitary-adrenal axis dysregulation accompanying sepsis may also increase the vulnerability to mood and anxiety disorders (18). In parallel, sepsis-related endothelial or vascular dysfunction and potential blood-brain barrier disturbance may promote neuroinflammation, offering a plausible biological pathway (18, 19). In a qualitative study, sepsis patients reported that the most influential factors of daily life to be the experience of psychologic and cognitive impairments including fatigue, as these often led to changes in social connections and everyday tasks: they often suffered from anxiety related to thoughts of death and fear of relationship changes (20).

The increased risk and high prevalence of psychiatric morbidity among sepsis patients underscores the need for integrated mental health screening and intervention strategies within post-sepsis care protocols. Our finding of a 43% cumulative risk at 5 years highlights the importance of close mental health monitoring and proactive support for patients and their families. Future research should explore effective strategies to support sepsis patients and reduce the onset of psychiatric morbidity.

A major strength of our study is the comprehensive inclusion of ICU-treated sepsis patients in Sweden, providing a large and representative sample. Additionally, the use of matched controls from the general population reduces confounding, and our robust adjustment for potential confounders using entropy balancing enhances the internal validity of our findings. Furthermore, the utilization of nationwide register data ensures comprehensive capture of diagnoses and prescription records, minimizing the risk of under-reporting.

Limitations include potential ascertainment bias, as sepsis patients likely have more healthcare contacts post-sepsis, increasing their likelihood of receiving psychotropic prescriptions. Also, reliance on national databases may miss psychiatric symptoms not captured in specialist care, and while inclusion of prescriptions of psychotropic medications helps mitigate this risk, some psychiatric conditions managed solely in primary care settings may still be under-reported. We assessed incident psychiatric events only, meaning that persistence, recurrence, severity, treatment duration, and functional dependency were not captured. Our outcome excluded delirium and other organic or cognitive syndromes typical of post intensive care syndrome. Thus, our results pertain to primary mood and anxiety disorders rather than acute organic states. The cohort comprised ICU-treated sepsis only, a more severely ill subgroup, meaning that findings may not generalize to sepsis managed on wards or intermediate care. Overall, results are most generalizable to settings similar to Sweden with high access to healthcare and comparable ICU case mix.

## CONCLUSIONS

Our findings show that patients treated in the ICU with sepsis face a higher risk of new psychotropic prescriptions or diagnoses of mood or anxiety disorders compared with matched controls from the general population, and that this elevated incidence can persist for up to 5 years after sepsis. Identifying patients at increased risk of psychiatric morbidity is important, and further research should concentrate on strategies to mitigate this long-term burden.

## ACKNOWLEDGMENTS

We thank Dr. Sten Walther, Swedish Intensive Care Registry (SIR) and Linköping University Hospital, Sweden, for the thoughtful advice and help extracting data from SIR.

## Supplementary Material
