# Supplementary material for: Sepsis and Subsequent Psychiatric Morbidity: A Nationwide Population-Based Matched Cohort Study, 2008–2019
Source: Crit Care Med. 2026 Mar 23;54(7):1527–33. doi: 10.1097/CCM.0000000000007105 (PMC13322153; doi:10.1097/CCM.0000000000007105)
Supplement: Supplementary file 1 [file ccm-54-1527-s001.pdf]

Long-Term Psychiatric Sequelae in Sepsis Survivors: A Nationwide Population-Based Matched Cohort Study

Supplementary material

Hanna Wetterberg, Anton Nilsson, Adam Linder, Maria Lengquist, Attila Frigyesi, Jonas Sundén-Cullberg, Malin Inghammar

Contents

Supplementary Methods 3
Supplementary Methods 1. 3
Supplementary Table 1. Specific codes for infectious disease 3
Supplementary Methods 2. STROBE Statement—Checklist of items that should be included in reports of cohort studies 4
Supplementary Table 2. Variables included in the entropy balancing 7
Supplementary Table 3. Specific codes for chronic diseases as mediators 10
Supplementary Results 11
Supplementary Table 4. Characteristics of the population before reweighting of the controls 11
Supplementary Table 5. Details of risk time and number of psychiatric events 13
Supplementary Figure 1. Weighted Kaplan-Meier curves of event-free survival for psychiatric events 14
Supplementary Table 6. Cumulative absolute risks and risk differences 15
Supplementary Table 7. Percentages of psychiatric events after the index date 16
Supplementary Figure 2. Landmark Cox regressions stratified by source of information (new prescription or diagnosis) 17
Supplementary Figure 3. Effect modification of the sepsis-psychiatric event association by sex, age, disease severity, treatment decision, and length of hospital stay 18
Supplementary Figure 4. Mediation of the sepsis-psychiatric morbidity association by new chronic diseases 21
References 22

Supplementary Methods

Supplementary Methods 1.

We defined community-acquired sepsis as any ICU admission with a main or secondary sepsis diagnosis, or an infection as the main diagnosis, occurring <2 days after arrival to the emergency department or hospital ward. We further required that patients had no prior surgical procedure recorded in the SIR and no hospital admission 3-30 days before the index date, using only the first sepsis episode for individuals with multiple events (see Supplementary Table 1 for ICD codes applied). The ICD-based identification of sepsis has been validated against the Sepsis-3 consensus criteria, showing an estimated accuracy of 83% [1].

Supplementary Table 1. Specific codes for infectious disease

Disease	ICD 10-code
Enteric infection	A00-A09
Sepsis (bloodstream infections)	A40-A41, R57.2, R65.0-1
Sexually transmitted infections	A50-A64, B20-B24
Infections of the neurologic system, including the eye	A39, A80-89, B30, G00-02, G03.9, G40.0-2, G04.9, G05-08, G94.0, H00.0, H03, H04.3, H05.0, H06.1, H10.0, H10.2-3, H10.9, H13.0-1, H16.2, H16.8-9, H19.0-2, H22.0, H32.0, H44.0, H45.1
Upper respiratory tract, including the ear	H60.0-3, H60.8-9, H61.0, H62, H66-67, H70, H73.0, H75.0, H83.0, H94.0, J00-06, J34.0, J36, J39.0-1
Lower respiratory tract infections, including influenza	A15-19, A48.1, J09-22, J44.0-1, J47, J69.0, J85-86
Infections of the heart and blood vessels	I30.1, I32.0-1, I33, I38-39, I40.0, I41.0-2, I43.0, I52.0-1, I68.1, I79.0-1, I98.0-1
Infections of the digestive system including the liver (narrow definition)	B15-19, K04.4, K04.6-7, K10.2, K11.3, K12.2, K23.0-1, K61, K63.0, K65.0, K67, K75.0, K77.0, K83.0, K93.0, K93.1
Infections of the genitourinary system	N08.0, N08.8, N10, N13.6, N15.1, N15.9, N16.0, N29.0-1, N30.0, N30.9, N33.0, N34.0-1, N37, N39.0, N41.0, N41.2-3, N43.1, N45.0, N45.9, N48.1-2, N49, N51, N61, N70-74, N75.1, N76.4, N77.0-1
Infections of the skin and soft tissue	A46, L00-03, L05.0, L08, L30.3
Infections of bone, joints and connective tissue	M00-01, M46.2-5, M49.0-2, M60.0, M63.0-2, M65.0, M68.0, M71.0-1, M72.5-6, M73.0-1, M86, M90.0-2
Infectious complications	T79.3, T80.2, T81.4, T82.6-7, T83.5-6, T84.5-7, T85.7, T87.4, T88.0
Other infections	A20-28, A30-38, A42-49, A65-69, A70-79, A92-99, B00-09, B25-27, B33-99, D70.9, D73.3, E06.0, E32.1
Note. Classification according to: Gustav T *et al.* Hospitalisations with infectious disease diagnoses in somatic healthcare between 1998 and 2019: A nationwide, register-based study in Swedish adults. *Lancet Reg Health Eur*. 2022;16:100343	

Supplementary Methods 2. STROBE Statement—Checklist of items that should be included in reports of cohort studies

	Item No	Recommendation	Page No
**Title and abstract**	1	(*a*) Indicate the study's design with a commonly used term in the title or the abstract<br><br>(*b*) Provide in the abstract an informative and balanced summary of what was done and what was found	1<br><br>2
**Introduction**			
Background/rationale	2	Explain the scientific background and rationale for the investigation being reported	4
Objectives	3	State specific objectives, including any prespecified hypotheses	4
**Methods**			
Study design	4	Present key elements of study design early in the paper	5
Setting	5	Describe the setting, locations, and relevant dates, including periods of recruitment, exposure, follow-up, and data collection	5
Participants	6	(*a*) Give the eligibility criteria, and the sources and methods of selection of participants. Describe methods of follow-up<br><br>(*b*) For matched studies, give matching criteria and number of exposed and unexposed	5, Figure 1
Variables	7	Clearly define all outcomes, exposures, predictors, potential confounders, and effect modifiers. Give diagnostic criteria, if applicable	6, 7, supplementary table 1, supplementary table 2, supplementary table 3

Data sources/ measurement	8*	For each variable of interest, give sources of data and details of methods of assessment (measurement). Describe comparability of assessment methods if there is more than one group	6
Bias	9	Describe any efforts to address potential sources of bias	6
Study size	10	Explain how the study size was arrived at	5, figure 1
Quantitative variables	11	Explain how quantitative variables were handled in the analyses. If applicable, describe which groupings were chosen and why	6
Statistical methods	12	(*a*) Describe all statistical methods, including those used to control for confounding<br>(*b*) Describe any methods used to examine subgroups and interactions<br>(*c*) Explain how missing data were addressed<br>(*d*) If applicable, explain how loss to follow-up was addressed<br>(*e*) Describe any sensitivity analyses	6, 7
**Results**			
Participants	13*	(a) Report numbers of individuals at each stage of study—eg numbers potentially eligible, examined for eligibility, confirmed eligible, included in the study, completing follow-up, and analysed<br>(b) Give reasons for non-participation at each stage<br>(c) Consider use of a flow diagram	5, figure 1
Descriptive data	14*	(a) Give characteristics of study participants (eg demographic, clinical, social) and information on exposures and potential confounders<br>(b) Indicate number of participants with missing data for each variable of interest<br>(c) Summarise follow-up time (eg, average and total amount)	7, table 1
Outcome data	15*	Report numbers of outcome events or summary measures over time	8, supplementary table 5

Main results	16	(*a*) Give unadjusted estimates and, if applicable, confounder-adjusted estimates and their precision (eg, 95% confidence interval). Make clear which confounders were adjusted for and why they were included<br><br>(*b*) Report category boundaries when continuous variables were categorized<br><br>(*c*) If relevant, consider translating estimates of relative risk into absolute risk for a meaningful time period	8, supplementary table 6, supplementary table 7
Other analyses	17	Report other analyses done—eg analyses of subgroups and interactions, and sensitivity analyses	8, 9, supplementary figure 2, supplementary figure 3, supplementary figure 4
**Discussion**			
Key results	18	Summarise key results with reference to study objectives	9, 10
Limitations	19	Discuss limitations of the study, taking into account sources of potential bias or imprecision. Discuss both direction and magnitude of any potential bias	11
Interpretation	20	Give a cautious overall interpretation of results considering objectives, limitations, multiplicity of analyses, results from similar studies, and other relevant evidence	12
Generalisability	21	Discuss the generalisability (external validity) of the study results	12
**Other information**			
Funding	22	Give the source of funding and the role of the funders for the present study and, if applicable, for the original study on which the present article is based	Title page

*Give information separately for exposed and unexposed groups.

Note: An Explanation and Elaboration article discusses each checklist item and gives methodological background and published examples of transparent reporting. The STROBE checklist is best used in conjunction with this article (freely available on the Web sites of PLoS Medicine at http://www.plosmedicine.org/, Annals of Internal Medicine at http://www.annals.org/, and Epidemiology at http://www.epidem.com/). Information on the STROBE Initiative is available at http://www.strobe-statement.org.

Supplementary Table 2. Variables included in the entropy balancing

**Medical history < 5 years of index**<br>**Main and additional discharge diagnoses**<br>**Inpatient and Outpatient Registry**	**ICD-10-SE[1], ATC[2], or procedure[3] code (ICD-10 codes unless otherwise specified)**
Enteric infection	A00-A09
Sepsis (bloodstream infections)	A40-A41, R57.2, R65.0-1
Sexually transmitted infections	A50-A64, B20-B24
Infections of the neurological system, including the eye	A39, A80-89, B30, G00-02, G03.9, G40.0-2, G04.9, G05-08, G94.0, H00.0, H03, H04.3, H05.0, H06.1, H10.0, H10.2-3, H10.9, H13.0-1, H16.2, H16.8-9, H19.0-2, H22.0, H32.0, H44.0, H45.1
Upper respiratory tract, including the ear	H60.0-3, H60.8-9, H61.0, H62, H66-67, H70, H73.0, H75.0, H83.0, H94.0, J00-06, J34.0, J36, J39.0-1
Lower respiratory tract infections, including influenza	A15-19, A48.1, J09-22, J44.0-1, J47, J69.0, J85-86
Infections of the heart and blood vessels	I30.1, I32.0-1, I33, I38-39, I40.0, I41.0-2, I43.0, I52.0-1, I68.1, I79.0-1, I98.0-1
Infections of the digestive system including the liver (narrow definition)	B15-19, K04.4, K04.6-7, K10.2, K11.3, K12.2, K23.0-1, K61, K63.0, K65.0, K67, K75.0, K77.0, K83.0, K93.0, K93.1
Infections of the genitourinary system	N08.0, N08.8, N10, N13.6, N15.1, N15.9, N16.0, N29.0-1, N30.0, N30.9, N33.0, N34.0-1, N37, N39.0, N41.0, N41.2-3, N43.1, N45.0, N45.9, N48.1-2, N49, N51, N61, N70-74, N75.1, N76.4, N77.0-1
Infections of the skin and soft tissue	A46, L00-03, L05.0, L08, L30.3
Infections of bone, joints and connective tissue	M00-01, M46.2-5, M49.0-2, M60.0, M63.0-2, M65.0, M68.0, M71.0-1, M72.5-6, M73.0-1, M86, M90.0-2
Other infections	A20-28, A30-38, A42-49, A65-69, A70-79, A92-99, B00-09, B25-27, B33-99, D70.9, D73.3, E06.0, E32.1
Acute coronary syndrome (MI)	I20.0, I21-22, I23
Other ischemic heart disease	I20.1-9, I24-25
Heart failure	I11.0, I13.0, I13.2, I42.0-5, I42.7-9. I.43, I50, I51.7, J81, K76.1
Valve disorders	I05-I09, I34-37
Other heart disease, hypertonia, cardiac surgery	I10, I11.9, I12.9, I13.9, I15, I27.1, I27.9, I30.0, I30.8-9, I51.0-6, I51.8-9
Vascular disease	I65, I70-72, I73.1, I73.8-9, I74, I77.1, I77.6, I79.0, I79.2, K55, I87.2, I89.0, I97.2, R02, Z95.1-9
Cerebrovascular disease	G45-46, I60-64, I67, I69
Thromboembolic disease	I26, I82
Arrhythmia	I44-49 (*not included*: I46.1), R00, Z45.0, Z95.0, T82.1

Pulmonary disease	E84, I27.0, I27.2-9, I28.0, I28.8-9, J41-47, J60-J67, J68.4, J70.1, J70.3, J80, J84, J92, J95, J96, J98.2, R09.2, Z99.2
Rheumatic disease	I00-02, J99.0-1, M05-09, M12.3, M13, M30, M31.1, M31.3-9, M32-34, M35.0-3, M35.8-9, M45-46, D86
Dementia	F00-03, F05.1, G30, G31.1, G31.8-9, G32
Hemiplegia, tetraplegia	G11.4, G80-82, G83.0-3, G83.8
Neurologic disease	G10-14, G20-21, G23, G35-37, G60-62, G70-71, G91, G93.1
Schizophrenia, bipolar disorder	F20-31
Other psychiatric disease	F04, F05.0, F05.8-9. F06-07, F09, F33-39, F44.9,
Drug or alcohol abuse, incl. intoxication	F10-19, K29.2, K70.0-1, G31.2, G62.1, G72.1, I42.6, R78.1-5, T40, T51, Z71.4-5
Diabetes	E10-14, I79.2
Kidney disease	N00-01, N03-07, N11-12, N17-19, N25.0, N26-27, N28.0, I12.0, I13.1, Q61.1-4, Z49, Z99.2, *Procedure codes:* DR016, DR024
Liver disease	K70.2-4, K70.9, K71.1, K71.3-5, K71.7, K72-74, K75.2-9, K76.0, K76.2-9, R18, I81, I85, I86.4, I98.2, I98.8
Gastro-intestinal disease	K50-52
Cancer, neoplasms	C00-D48, J70.0, T45.1, Z51.0, Z51.1 (*not included*: Z85)
HIV/AIDS	B20-24, F02.4, O98.7, R75, Z11.4, Z21.9, Z71.1
Immune deficiency, blood disease, anaemia	D50.1-9, D51-53, D60-61, D64.9, D65-68, D69.1, D69.3-6, D70-72, D73.0-2, D73.5-9, D76, D80-84, D89
Other conditions	G96.0, E22.2, E40-44, E46, E64, E66, E86, E87, R40.2, R64, R63.4
**Medical history at any time < 5 > years**<br>**Main and additional discharge diagnoses**<br>**Inpatient and Outpatient Registry**	
Any medical history	
Cardiac surgery	*Procedure codes:* F(A-X), I97.0-1,
Organ transplantation	Z94, T86, *Procedure codes:* KAS, FQA, FQB, JJC, GDG, JLE
Childhood conditions	F71.1, F72-73, F79.1, F83. F84, G80, Q01-03, Q05, Q20-26, Q90
**ATC-codes < 1 year**<br>**Drug prescription Registry**	
Cardiac disease	C09A-D, CCB, C08C, C08D, C03C, C03EB, C03A, C03B, C03D, C03EA, C07, C01B, C01AA05, C01DA, B01AC, B01AA, C10
Lung disease	R03AC, R03BB, R03BA, R03AK, R03DA
Diabetes	A10B, A10A
Rheumatic disease	M01A
Psychiatric disease	N05A, N05B, N05C, N06A, N06D, N07BB, N07BC
Immune suppressive drugs	H02AB, L01B, L04A, L01

**Health care use**	
No of hospitalizations in last 5 years	
No of days in hospital in last 5 years	
No of (all codes) hospitalizations in last year	
No of (all codes) outpatient visits in last year	
No of drug used in last year	
No of days of hospitalization for infectious diseases in last 5 years	
No of hospitalizations for infectious diseases in last 5 years	
No of hospitalizations in last 6 months	
**Sociodemographic factors**<br>**LISA registry[4]**	
Age	Age at admission
Sex	
County of residence	County of residence within Sweden
Region of birth	Scandinavia, rest of Europe, or rest of the world (including unknown)
Year of admission	Year of sepsis admission
Education	Primary, short secondary, long secondary, tertiary, or missing information
Occupational status	Unemployment, employment, sickness absence, or retirement. Based on main source of income the year before sepsis.
Disposable income	Disposable income the year before index date, quintiles

Notes.
[1] https://www.socialstyrelsen.se/statistik-och-data/klassifikationer-och-koder/icd-10/, accessed 2023-10-11
[2] https://www.whocc.no/atc_ddd_index/, accessed 2023-10-11
[3] https://www.socialstyrelsen.se/statistik-och-data/klassifikationer-och-koder/kva/, accessed 2023-10-11
[4] Longitudinal integrated databases for health insurance and labour market studies

Supplementary Table 3. Specific codes for chronic diseases as mediators

**Chronic disease**	**ICD-10 codes**
Chronic pain syndromes:<br>Pain, not elsewhere classified	R52
Neurological complications:<br>polyneuropathy, unspecified, Encephalopathy, unspecified	G62.9, G93.4
Cardiovascular complications:<br>Heart failure, Sequelae of cerebrovascular disease	I50, I69
Respiratory conditions:<br>Respiratory failure, other disorders of lung	J96, J98.4
Renal failure:<br>Acute and chronic renal failure	N17-N19
Note. Conditions listed were treated as time-varying mediators and had to be newly diagnosed after the index (sepsis) date and before the psychiatric event or censoring. Ascertainment used ICD-10 codes from the National Patient Register (inpatient and specialist outpatient care). Only the first occurrence per mediator was used.	

Supplementary Results

Supplementary Table 4. Characteristics of the population before reweighting of the controls

Variable	Sepsis patients (n=10 308)	Controls, unweighted (n=155 705)
Basic sociodemographics		
Median and mean age (years)	70.0; 66.8 [sd: 15.7]	69.0; 65.3 [sd: 15.9]
Female sex, % (n)	37.6 (3875)	34.0 (53011)
Place of birth		
Nordic country, % (n)	92.4 (9520)	89.9 (140028)
Non-Nordic European country, % (n)	3.6 (374)	5.4 (8469)
Non-European country, % (n)	4.0 (414)	4.6 (7208)
Education		
Missing information, % (n)	2.2 (223)	1.8 (2734)
Primary education, % (n)	38.2 (3941)	29.9 (46553)
Short secondary education, % (n)	28.4 (2927)	26.1 (40658)
Long secondary education, % (n)	12.9 (1335)	15.2 (23655)
Tertiary education, % (n)	18.3 (1882)	27.0 (42105)
Employment status		
Employed, % (n)	25.4 (2617)	35.6 (55515)
Retired, % (n)	61.5 (6337)	56.8 (88451)
Sickness absence, % (n)	1.6 (162)	0.5 (835)
Unemployed, % (n)	11.6 (1192)	7.0 (10904)
Disposable income		
Quintile 1, % (n)	25.5 (2626)	17.8 (27685)
Quintile 2, % (n)	22.6 (2329)	18.3 (28525)
Quintile 3, % (n)	20.1 (2071)	20.4 (31706)
Quintile 4, % (n)	17.3 (1782)	21.6 (33712)
Quintile 5, % (n)	14.6 (1500)	21.9 (34077)
Inpatient visits		
Median and mean visits, past 5 years	1.0; 2.0 [sd: 3.0]	0.0; 0.7 [sd: 1.4]
Median and mean visits, past year	0.0; 0.6 [sd: 1.1]	0.0; 0.1 [sd: 0.5]
Median and mean days, past 5 years	4.0; 15.5 [sd: 30.7]	0.0; 3.6 [sd: 11.4]
Median and mean days, past year	0.0; 4.6 [sd: 12.0]	0.0; 0.7 [sd: 3.8]
Outpatient visits		
Median and mean outpatient visits, past year	1.0; 3.1 [sd: 7.6]	0.0; 1.2 [sd: 3.1]
Pharmaceuticals		
Median and mean types of drugs, past year	4.0; 4.7 [sd: 4.0]	2.0; 2.6 [sd: 2.8]
Inpatient visits due to infectious disease		
Median and mean visits, past 5 years	0.0; 0.3 [sd: 0.9]	0.0; 0.0 [sd: 0.3]
Median and mean days, past 5 years	0.0; 2.8 [sd: 10.9]	0.0; 0.3 [sd: 2.8]
Specific diagnoses or procedures, past 5 years		
Acute coronary syndrome, % (n)	4.0 (414)	2.1 (3317)
Other ischemic heart disease, % (n)	0.1 (11)	0.1 (80)
Heart failure/cardiomyopathy, % (n)	12.2 (1255)	3.0 (4699)
Valve disorders, % (n)	4.4 (450)	1.9 (2933)
Other heart diseases, hypertonia, or cardiac surgery, % (n)	0.4 (37)	0.1 (193)
Vascular disease, % (n)	13.0 (1344)	6.2 (9623)

Cerebrovascular disease, % (n)	5.7 (591)	3.3 (5195)
Thromboembolic disease, % (n)	2.4 (245)	0.7 (1112)
Arrythmia, % (n)	18.9 (1948)	8.8 (13781)
Pulmonary disease, % (n)	11.3 (1169)	3.5 (5402)
Rheumatic disease, % (n)	4.7 (482)	1.6 (2550)
Dementia, % (n)	1.0 (102)	0.7 (1116)
Hemiplegia or tetraplegia, % (n)	1.0 (102)	0.1 (177)
Neurologic disease, % (n)	3.7 (381)	1.1 (1727)
Schizophrenia or bipolar disorder, % (n)	0.2 (20)	0.1 (99)
Other psychiatric disorder, % (n)	0.7 (76)	0.4 (572)
Drug, alcohol abuse, or intoxication, % (n)	5.3 (548)	1.2 (1827)
Diabetes, % (n)	18.7 (1926)	6.7 (10364)
Kidney disease, % (n)	8.3 (853)	1.6 (2473)
Liver disease, % (n)	1.5 (150)	0.1 (199)
Gastro-intestinal disease, % (n)	2.7 (278)	1.2 (1807)
Neoplasms, % (n)	24.5 (2529)	15.8 (24616)
HIV/AIDS, % (n)	0.2 (20)	0.1 (79)
Immune deficiency, blood disease, or anemia, % (n)	10.1 (1043)	2.1 (3200)
Other (non-infectious) diseases, % (n)	9.3 (959)	2.0 (3159)
Enteric infection, % (n)	3.3 (336)	0.9 (1390)
Sepsis, % (n)	5.8 (595)	0.7 (1031)
Infectious of the neurological system, including the eye, % (n)	0.4 (38)	0.1 (221)
Upper respiratory tract infection, including the ear, % (n)	2.8 (292)	1.5 (2290)
Lower respiratory tract infection, including influenza, % (n)	13.2 (1357)	3.3 (5120)
Infection of the heart or blood vessels, % (n)	0.3 (34)	0.1 (114)
Infection of the digestive system, including the liver, % (n)	1.8 (181)	0.4 (555)
Infection of the genitourinary system, % (n)	12.1 (1248)	3.4 (5333)
Infections of the skin or soft tissue, % (n)	6.7 (691)	1.4 (2256)
Infection of bone, joints, or connective tissue, % (n)	0.9 (94)	0.2 (354)
Other infections, % (n)	15.8 (1632)	4.3 (6757)
Specific diagnoses, ever		
Cardiac surgery, % (n)	10.7 (1101)	7.1 (11018)
Organ transplantation, % (n)	1.9 (200)	0.3 (425)
Childhood conditions, % (n)	1.1 (111)	0.3 (472)
Specific pharmaceuticals, last year		
Cardiac disease, % (n)	63.4 (6532)	47.8 (74440)
Lung disease, % (n)	14.4 (1479)	7.9 (12330)
Diabetes, % (n)	20.2 (2079)	9.4 (14621)
Rheumatic disease, % (n)	13.7 (1410)	11.9 (18478)
Dementia, alcohol, or opioid dependence % (n)	1.1 (108)	0.9 (1319)
Immunosuppressive drugs, % (n)	18.0 (1853)	5.5 (8534)
Outcome information (general)		
Median and mean follow-up time (years)	0.7; 2.1 [sd: 2.8]	3.6; 4.2 [sd: 3.1]
Died during follow-up, % (n)	48.2 (4972)	11.6 (18018)

The table shows descriptive statistics before reweighting the controls. Background characteristics refer to the status at the index date. Quintiles of disposable household income were defined according to the distribution among controls. Sd: Standard deviation

Supplementary Table 5. Details of risk time and number of psychiatric events

Details of risk time and number of psychiatric events and observations								
	Number at risk		Number of events		Total observation time (years)		Incidence rates (1000/PY)	
Outcome	Cases	Controls	Cases	Controls	Cases	Controls	Cases	Controls
**Any psychiatric event**								
Total follow-up	10 308	10 308	3 101	2 634	21 336	38 472	145.3	68.5
0-30 days	10 308	10 308	474	96	691	838	685.5	114.7
31-90 days	7 469	7 469	755	110	1 103	1 205	684.8	91.2
91-365 days	6 181	6 181	733	344	3 992	4 279	183.6	80.3
1-3 years	4 651	4 651	616	517	7 228	7 492	85.2	69.0
3-5 years	2 722	2 722	310	246	4 199	4 333	73.8	56.8
≥5 years	1 552	1 552	213	199	4 120	4 190	51.7	47.6
**New prescription**								
Total follow-up	10 308	10 308	3 060	2 594	21 336	38 472	143.4	67.4
0-30 days	10 308	10 308	446	94	693	838	643.7	112.4
31-90 days	7 496	7 514	748	108	1 108	1 212	675.2	89.0
91-365 days	6 213	6 225	731	338	4 014	4 312	182.1	78.5
1-3 years	4 651	4 651	608	508	7 228	7 492	84.1	67.8
3-5 years	2 722	2 722	306	242	4 199	4 333	72.9	55.8
≥5 years	1 552	1 552	210	194	4 120	4 190	51.0	46.4
**New diagnosis**								
Total follow-up	10 308	10 308	337	243	29 743	45 263	11.3	5.4
0-30 days	10 308	10 308	38	3	704	842	54.0	4.1
31-90 days	7 897	7 895	42	7	1 236	1 282	34.0	5.8
91-365 days	7 261	7 260	67	30	4 985	5 145	13.4	5.8
1-3 years	6 069	6 068	84	57	9 885	10 256	8.5	5.5
3-5 years	3 965	3 965	48	36	6 341	6 558	7.6	5.5
≥5 years	2 440	2 440	58	37	6 588	6 930	8.8	5.4

Note. Control counts and person-time are weighted using doubly robust entropy balancing; weighted totals were rounded to the nearest integer. PY = person years.

Supplementary Figure 1. Weighted Kaplan-Meier curves of event-free survival for psychiatric events

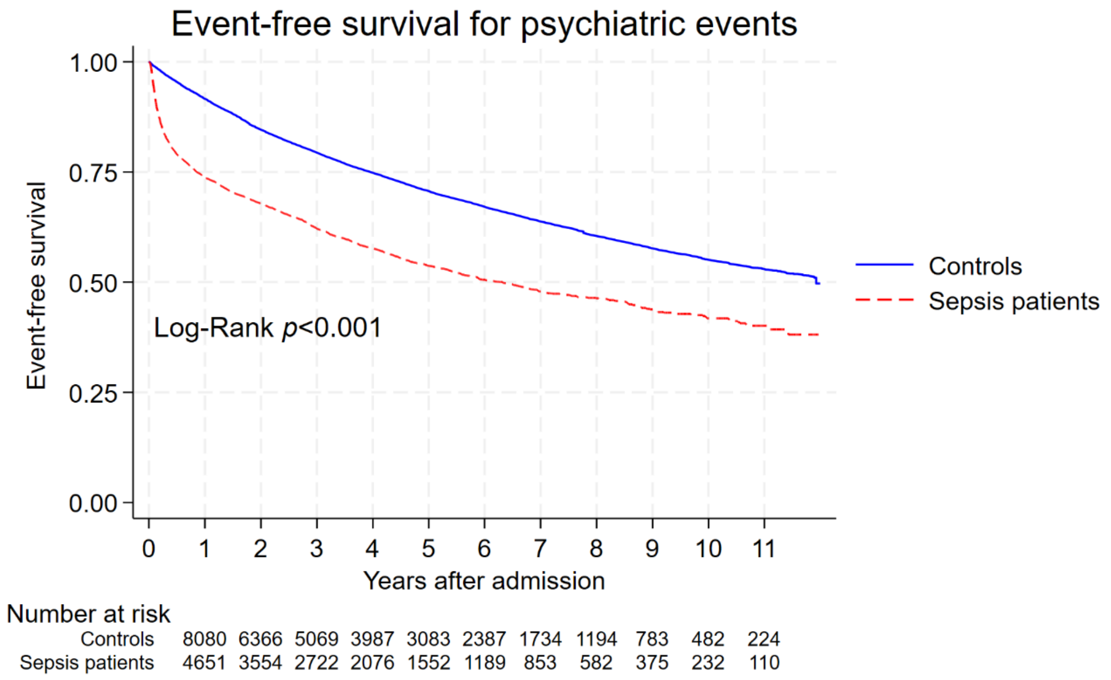

Weighted Kaplan-Meier curves of event-free survival for psychiatric event after the index date. Curves and at-risk counts are weighted using entropy balancing weights applied to controls; displayed counts are rounded.

Supplementary Table 6. Cumulative absolute risks and risk differences

Absolute risks and risk differences for psychiatric events									
Follow-up	Controls, %	Cases, %	Difference, %	Controls, %	Cases, %	Difference, %	Controls, %	Cases, %	Difference, %
**Total**									
At 31 days	1.0	5.9	5.0						
At 91 days	2.5	15.7	13.2						
At 1 year	8.4	26.2	17.9						
At 3 years	20.6	37.8	17.2						
At 5 years	29.3	46.3	17.0						
**Sex**		**Males**			**Females**				
At 31 days	1.0	5.8	4.8	0.9	6.1	5.3			
At 91 days	2.4	15.4	13.0	2.6	16.2	13.6			
At 1 year	8.1	25.7	17.7	8.9	27.0	18.1			
At 3 years	19.9	37.6	17.7	21.7	38.2	16.5			
At 5 years	28.4	45.8	17.4	30.8	47.1	16.3			
**Age Group**		**<65**			**≥65**				
At 31 days	0.8	4.9	4.1	1.0	6.6	5.6			
At 91 days	1.9	14.0	12.0	2.8	16.9	14.1			
At 1 year	5.9	24.1	18.2	9.8	27.6	17.9			
At 3 years	14.2	34.2	20.0	24.4	40.6	16.2			
At 5 years	20.3	41.0	20.7	35.0	50.7	15.7			
**SAPS3 level**		**Level 1**			**Level 2**			**Level 3**	
At 31 days	0.8	4.7	3.8	0.8	6.9	6.1	1.1	7.2	6.1
At 91 days	2.1	11.7	9.7	2.3	17.2	14.9	2.9	22.9	20.0
At 1 year	7.2	21.0	13.8	8.6	28.1	19.5	9.5	35.6	26.0
At 3 years	17.2	32.3	15.1	21.7	41.4	19.7	23.9	46.0	22.0
At 5 years	25.0	39.8	14.8	31.3	50.7	19.4	33.4	56.7	23.2

Note. Estimates are weighted using entropy balancing applied to controls. SAPS3: Simplified Acute Physiology Score version 3. Survival analysis conducted on cohort data, including cases and controls. Percentages are rounded to one decimal place.

Supplementary Table 7. Percentages of psychiatric events after the index date

**Variable**	**Sepsis patients, n (%) (N=10 308)**	**Controls, crude, n (%) (N=155,705)**	**Controls, weighted, n (%) (N=10 308)**
Any psychiatric event	3101 (30.1)	32,191 (20.7)	2634 (25.6)
Prescription			
Any psychiatric medication	3060 (29.7)	31,614 (20.3)	2593 (25.2)
Antipsychotics	275 (2.7)	2931 (1.9)	248 (2.4)
Anxiolytics	1023 (9.9)	10,929 (7.0)	934 (9.1)
Hypnotics and sedatives	2046 (19.9)	18,615 (12.0)	1479 (14.4)
Antidepressants	1472 (14.3)	15,831 (10.2)	1305 (12.7)
Diagnosis from inpatient or outpatient specialists care			
Any psychiatric diagnosis	337 (3.3)	3040 (2.0)	243 (2.4)
Mood disorders (incl depressive episodes)	194 (1.9)	1470 (0.9)	124 (1.2)
Anxiety disorders	201 (2.0)	2069 (1.3)	156 (1.5)

Note. Counts and percentages of psychiatric events after index date among sepsis cases, crude controls, and weighted controls (entropy balancing applied to controls). Estimates represent the proportion experiencing ≥1 event over the entire follow-up (index date to event, death, emigration or study end). Values rounded to one decimal.

Supplementary Figure 2. Landmark Cox regressions stratified by source of information (new prescription or diagnosis)

Time intervals to psychiatric event	Number of events: Controls No. of events/ No. at risk (%)	Number of events: Sepsis cases No. of events/ No. at risk (%)	aHR, 95% CI
**New prescription**			
Days 0-30	94/10 308 (0.9)	446/10 308 (4.3)	5.9 (4.7, 7.4)
Days 31-90	108/7514 (1.4)	748/7496 (10.0)	7.5 (6.5, 8.7)
Days 91-365	338/6225 (5.4)	731/6213 (11.8)	2.3 (2.1, 2.5)
Years >1-3	508/4651 (10.9)	608/4651 (13.1)	1.2 (1.1, 1.4)
Years >3-5	242/2722 (8.9)	306/2722 (11.2)	1.3 (1.2, 1.5)
Years ≥5	194/1552 (12.5)	210/1552 (13.5)	1.1 (0.9, 1.3)
**New diagnosis**			
Days 0-30	3/10 308 (0.0)	38/10 308 (0.4)	13.1 (7.5, 23.1)
Days 31-90	7/7895 (0.1)	42/7897 (0.5)	5.8 (2.7, 12.6)
Days 91-365	30/7260 (0.4)	67/7261 (0.9)	2.3 (1.7, 3.2)
Years >1-3	57/6068 (0.9)	84/6069 (1.4)	1.5 (1.2, 2.0)
Years >3-5	36/3965 (0.9)	48/3965 (1.2)	1.4 (1.0, 1.9)
Years ≥5	37/2440 (1.5)	58/2440 (2.4)	1.6 (1.2, 2.2)

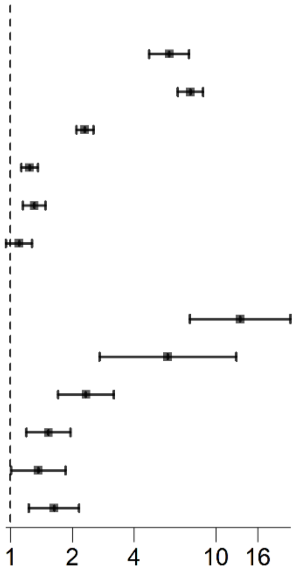

Note. aHR=Adjusted hazard ratios for new prescription or diagnosis in sepsis cases versus matched,weighted controls, estimated using Cox regression. Control numbers are rounded. Weights were calculated using individuals eligable for each landmark period. The figure uses a log scale

Supplementary Figure 3. Effect modification of the sepsis-psychiatric event association by sex, age, disease severity, treatment decision, and length of hospital stay

a)

Time intervals to psychiatric event	Controls No. of events/ No. at risk (%)		Sepsis cases No. of events/ No. at risk (%)		aHR, 95% CI
0-30 Days	33/3875	(0.8)	183/3875	(4.7)	7.1 (5.4, 9.2)
	63/6433	(1.0)	291/6433	(4.5)	5.7 (4.2, 7.7)
31-90 Days	48/2805	(1.7)	294/2805	(10.5)	6.6 (5.2, 8.3)
	62/4664	(1.3)	461/4664	(9.9)	8.0 (6.7, 9.6)
91-365 Days	147/2328	(6.3)	287/2328	(12.3)	2.1 (1.7, 2.4)
	203/3853	(5.3)	446/3853	(11.6)	2.4 (2.1, 2.7)
>1-3 Years	215/1774	(12.1)	234/1774	(13.2)	1.1 (1.0, 1.3)
	301/2877	(10.5)	382/2877	(13.3)	1.3 (1.2, 1.5)
>3-5 Years	104/1070	(9.7)	129/1070	(12.1)	1.3 (1.1, 1.6)
	142/1652	(8.6)	181/1652	(11.0)	1.3 (1.1, 1.6)
≥5 Years	87/608	(14.2)	87/608	(14.3)	1.0 (0.8, 1.3)
	113/944	(12.0)	126/944	(13.3)	1.1 (0.9, 1.4)

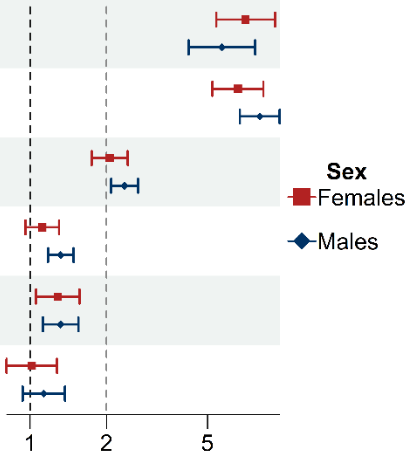

b)

Time intervals to psychiatric event	Controls No. of events/ No. at risk (%)		Sepsis cases No. of events/ No. at risk (%)		aHR, 95% CI
0-30 Days	28/3538	(0.8)	155/3557	(4.4)	6.2 (3.9, 9.8)
	68/6770	(1.0)	319/6751	(4.7)	6.3 (4.9, 8.0)
31-90 Days	41/2951	(1.4)	269/2951	(9.1)	6.9 (4.8, 10.1)
	74/4518	(1.6)	486/4518	(10.8)	7.2 (6.1, 8.4)
91-365 Days	109/2553	(4.3)	295/2553	(11.6)	2.9 (2.3, 3.5)
	234/3628	(6.4)	438/3628	(12.1)	2.0 (1.8, 2.2)
>1-3 Years	163/2030	(8.0)	241/2030	(11.9)	1.5 (1.3, 1.8)
	341/2621	(13.0)	375/2621	(14.3)	1.1 (1.0, 1.3)
>3-5 Years	83/1347	(6.2)	122/1347	(9.1)	1.5 (1.2, 1.9)
	157/1375	(11.4)	188/1375	(13.7)	1.2 (1.1, 1.4)
≥5 Years	92/877	(10.5)	111/877	(12.7)	1.2 (1.0, 1.5)
	108/675	(16.0)	102/675	(15.1)	0.9 (0.8, 1.2)

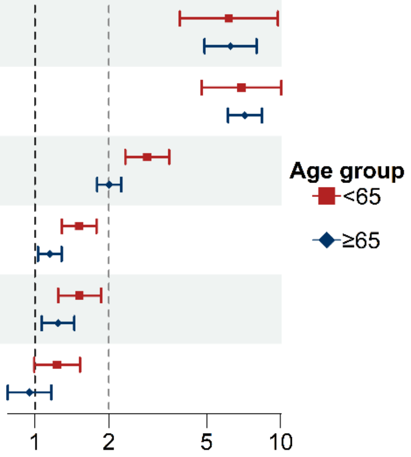

c)

Time intervals to psychiatric event	Controls No. of events/ No. at risk (%)		Sepsis cases No. of events/ No. at risk (%)		aHR, 95% CI
0-30 Days	30/3547	(0.8)	155/3656	(4.2)	5.4 (3.5, 8.4)
	25/3300	(0.8)	181/3352	(5.4)	8.6 (6.4, 11.7)
	30/2822	(1.1)	112/2672	(4.2)	6.6 (4.5, 9.6)
31-90 Days	39/3197	(1.2)	236/3197	(7.4)	6.3 (4.8, 8.2)
	38/2452	(1.6)	259/2452	(10.6)	7.4 (5.9, 9.2)
	21/1343	(1.6)	215/1343	(16.0)	11.8 (9.5, 14.5)
91-365 Days	142/2821	(5.0)	282/2821	(10.0)	2.1 (1.8, 2.5)
	121/1963	(6.2)	246/1963	(12.5)	2.2 (1.8, 2.6)
	65/990	(6.5)	149/990	(15.1)	2.5 (2.1, 3.1)
>1-3 Years	203/2232	(9.1)	275/2232	(12.3)	1.4 (1.2, 1.6)
	175/1418	(12.3)	214/1418	(15.1)	1.3 (1.1, 1.5)
	91/677	(13.4)	87/677	(12.9)	1.0 (0.8, 1.3)
>3-5 Years	106/1388	(7.6)	130/1388	(9.4)	1.2 (1.0, 1.5)
	80/745	(10.7)	94/745	(12.6)	1.2 (1.0, 1.5)
	36/343	(10.5)	56/343	(16.3)	1.6 (1.2, 2.2)
≥5 Years	92/827	(11.2)	94/827	(11.4)	1.0 (0.8, 1.3)
	53/376	(14.1)	65/376	(17.3)	1.3 (1.0, 1.7)
	23/158	(14.6)	13/158	(8.2)	0.5 (0.3, 0.9)

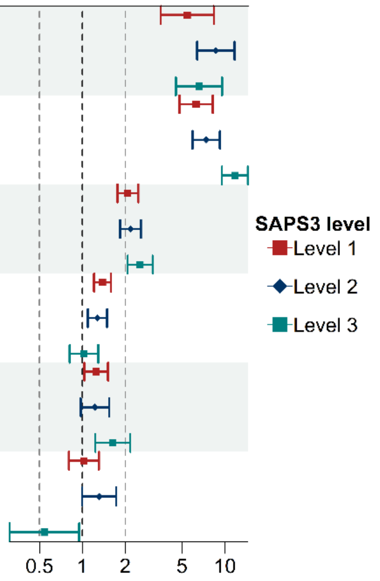

d)

Time intervals to psychiatric event	Controls No. of events/ No. at risk (%)		Sepsis cases No. of events/ No. at risk (%)		aHR, 95% CI
0-30 Days	56/5935	(0.9)	295/6172	(4.8)	5.7 (4.2, 7.8)
	30/2742	(1.1)	109/2870	(3.8)	5.6 (3.9, 8.1)
31-90 Days	66/5033	(1.3)	491/5033	(9.8)	8.0 (6.8, 9.4)
	23/1516	(1.5)	169/1516	(11.1)	8.3 (6.3, 11.0)
91-365 Days	224/4239	(5.3)	487/4239	(11.5)	2.3 (2.0, 2.6)
	77/1175	(6.5)	147/1175	(12.5)	2.1 (1.7, 2.5)
>1-3 Years	343/3241	(10.6)	417/3241	(12.9)	1.3 (1.1, 1.4)
	94/803	(11.7)	110/803	(13.7)	1.2 (1.0, 1.5)
>3-5 Years	176/1941	(9.1)	228/1941	(11.7)	1.3 (1.2, 1.5)
	24/339	(7.2)	31/339	(9.1)	1.3 (0.9, 1.9)
≥5 Years	150/1179	(12.7)	169/1179	(14.3)	1.2 (1.0, 1.4)

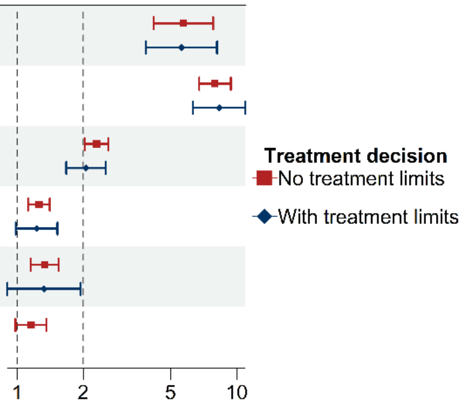

e)

Time intervals to psychiatric event	Controls No. of events/ No. at risk (%)		Sepsis cases No. of events/ No. at risk (%)		aHR, 95% CI
0-30 Days	58/5858	(1.0)	244/6033	(4.0)	5.1 (3.8, 6.9)
	18/2101	(0.8)	139/2241	(6.2)	9.2 (5.5, 15.6)
	20/2349	(0.9)	91/2034	(4.5)	6.4 (4.3, 9.6)
31-90 Days	71/4423	(1.6)	279/4423	(6.3)	4.1 (3.3, 5.0)
	21/1607	(1.3)	182/1607	(11.3)	9.3 (6.9, 12.6)
	16/1439	(1.1)	294/1439	(20.4)	21.5 (16.9, 27.4)
91-365 Days	211/3876	(5.4)	398/3876	(10.3)	2.0 (1.8, 2.3)
	75/1324	(5.6)	134/1324	(10.1)	1.9 (1.5, 2.3)
	52/981	(5.3)	201/981	(20.5)	4.4 (3.6, 5.3)
>1-3 Years	327/2931	(11.2)	385/2931	(13.1)	1.2 (1.1, 1.4)
	122/1042	(11.7)	142/1042	(13.6)	1.2 (1.0, 1.5)
	68/678	(10.0)	89/678	(13.1)	1.4 (1.1, 1.7)
>3-5 Years	156/1688	(9.3)	184/1688	(10.9)	1.2 (1.0, 1.4)
	57/611	(9.3)	73/611	(11.9)	1.3 (1.0, 1.8)
	37/423	(8.9)	53/423	(12.5)	1.5 (1.1, 2.0)
≥5 Years	124/955	(13.0)	133/955	(13.9)	1.1 (0.9, 1.3)
	48/344	(13.9)	46/344	(13.4)	1.0 (0.7, 1.4)
	33/253	(13.0)	34/253	(13.4)	1.1 (0.8, 1.5)

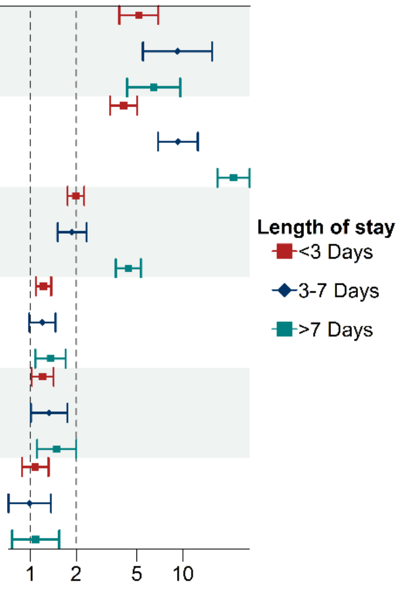

Note. aHR=Adjusted hazard ratios for psychiatric events (new prescription or diagnosis) in sepsis cases versus matched, weighted controls, estimated using Cox regression, stratified by a) sex, b) age group, c) SAPS3 category, d) treatment descision, and e) hospital length of stay. The figure uses a log scale

Supplementary Figure 4. Mediation of the sepsis-psychiatric morbidity association by new chronic diseases

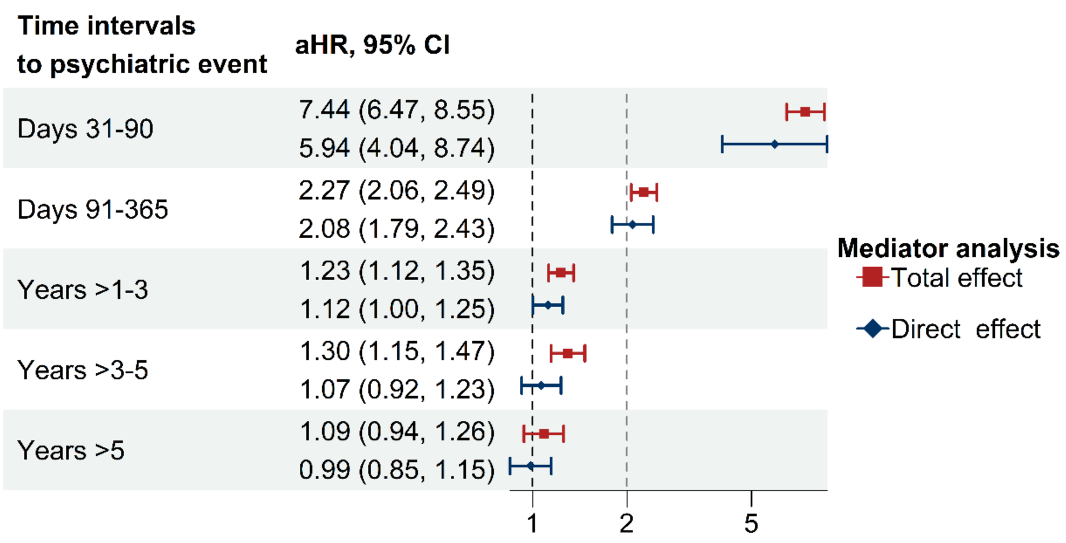

Note. aHR=Adjusted hazard ratios for psychiatric events (new prescription or diagnosis) in sepsis cases versus matched, weighted controls, estimated using Cox regression. The mediator could occur anytime between sepsis baseline and the end of each landmark, but prior to psychiatric event. Only the first event of the mediator was included.
The figure uses a log scale

References

1. Lengquist M, Lundberg OHM, Spangfors M: **Sepsis is underreported in Swedish intensive care units: A retrospective observational multicentre study (vol 64, pg 1167, 2020)**. *Acta Anaesth Scand* 2021, **65**(1):140–140.
